# Primary thyroid T‐cell lymphoma with leukemic manifestation

**DOI:** 10.1002/jha2.31

**Published:** 2020-06-28

**Authors:** Toru Kawakami, Hideyuki Nakazawa, Fumihiro Ishida

**Affiliations:** ^1^ Department of Internal Medicine Division of Hematology Shinshu University School of Medicine Matsumoto Japan; ^2^ Department of Health and Medical Sciences Graduate School of Medicine Shinshu University Matsumoto Japan; ^3^ Department of Biomedical Laboratory Sciences Shinshu University School of Medicine Matsumoto Japan

**FIGURE 1:**
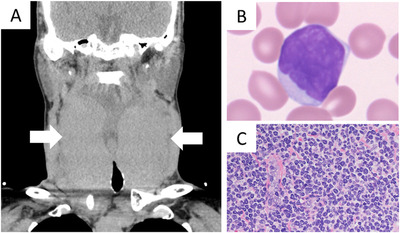
A, Computed tomography (coronal section). B, Wright‐Giemsa staining (original magnification ×100). C, Hematoxylin and eosin (H&E) staining (original magnification ×20)

A 63‐year‐old woman presented with asymptomatic giant goiter that has been unchanged in size for the preceding years (Figure 1A). She had 5‐year history of Hashimoto thyroiditis being controlled with levothyroxine. The blood tests revealed she had had persistently increased number of moderately large atypical lymphocytes, with scant cytoplasm and cleaved and wrinkled nuclei (Figure 1B). They were CD2^+^, CD3^+^, CD4^+^, and CD8^–^, and T cell receptor beta and gamma gene rearrangements were positive. Bone marrow aspirates showed 6% infiltrates of the identical lymphocytes, and its biopsy specimen also harbored sparse small aggregates. An incisional thyroid biopsy revealed diffuse infiltration of small‐to‐medium‐sized lymphoid cells (Figure 1C). Those cells were positive for CD3, CD4, CXCL13, CCR4, and FOXP3 and negative for CD56, TIA‐1, and granzyme B. Epstein‐Barr virus genome was negative with in situ hybridization method. The patient was tested negative for HTLV‐1 or HIV antibodies. Primary thyroid T‐cell lymphoma (PTTL) was diagnosed. After 18 months of watchful waiting, CHOP therapy was started. Six courses of the chemotherapy yielded partial response of the lymphoma.

PTTL is a provisional entity proposed as a subtype of peripheral T‐cell lymphoma [1]. Although a leukemic manifestation is often described at presentation as was in the present case, the clinical course is usually indolent.
